# Small Molecule Activators of Protein Phosphatase 2A Exert Global Stabilizing Effects on the Scaffold PR65

**DOI:** 10.1021/jacsau.6c00003

**Published:** 2026-04-20

**Authors:** Mohsin M. Naqvi, Maria Zacharopoulou, Satyaki Saha, Anupam Banerjee, Sema Z. Yilmaz, Vanda Sunderlikova, Chris M. Johnson, Janet R. Kumita, Shang-Hua Yang, Reuven Gordon, Michael Ohlmeyer, Sander J. Tans, Mert Gur, Ivet Bahar, Laura S. Itzhaki

**Affiliations:** † Department of Pharmacology, 2152 University of Cambridge, Tennis Court Road, Cambridge CB2 1PD, U.K.; ‡ Laufer Center for Physical and Quantitative Biology, Stony Brook University, Stony Brook, New York 11794, United States; § Department of Biochemistry and Cell Biology, Renaissance School of Medicine, Stony Brook University, Stony Brook, New York 11794, United States; ∥ Department of Computational and Systems Biology, School of Medicine, University of Pittsburgh, Pittsburgh, Pennsylvania 15260, United States; ⊥ Autonomous Matter Department, 55952 AMOLF institute, Science Park 104, Amsterdam 1098 XG, The Netherlands; # Department of Bionanoscience, Kavli Institute of Nanoscience Delft, Delft University of Technology, HZ Delft 2629, The Netherlands; ¶ 47694 MRC Laboratory of Molecular Biology, Francis Crick Avenue, Cambridge CB2 0QH, U.K.; ∇ Department of Electrical Engineering, 34881 National Tsing Hua University, Hsinchu 30013, Taiwan; ○ Department of Electrical and Computer Engineering, 8205 University of Victoria, Victoria, British Columbia V8P 5C2, Canada; ⧫ Atux Iskay LLC, Plainsboro, New Jersey NJ, 08536, United States

**Keywords:** PR65, PP2A, optical tweezers, HEAT repeat, SMAP, pharmacological chaperone

## Abstract

Protein phosphatase 2A (PP2A), an important therapeutic target, comprises scaffold subunit PR65 composed of 15 HEAT (Huntingtin/Elongation/A-subunit/TOR1) repeats, a catalytic subunit, and one of many different regulatory subunits that enable binding to specific substrates. Recently, small molecule activators of PP2A (SMAPs) were identified, although their mechanisms of action have not yet been fully defined. Here, we explore the interaction of PR65 with two SMAPs, ATUX-8385 and the nonfunctional DBK-776, using single-molecule optical tweezers, ensemble methods, and computational analysis. In the absence of SMAP, PR65 shows multiple unfolding and refolding transitions, and the force–extension profiles are very heterogeneous with evidence of misfolding. Similar heterogeneity has been observed for the chemical-induced unfolding of tandem-repeat proteins like PR65, a consequence of the internal symmetry of the repeat architecture. In the presence of ATUX-8385, higher unfolding and refolding forces are observed throughout the structure and there is less misfolding, suggesting that ATUX-8385 acts like a pharmacological chaperone. In contrast, DBK-766-binding induces higher unfolding forces only for a few repeats of PR65, suggestive of a more localized effect; moreover, subsequent stretch–relax cycles show that PR65 is irreversibly locked in the unfolded state. Docking and molecular dynamics simulations provide insights into the distinctive responses of PR65 to mechanical stress in the presence of these two SMAPs: ATUX-8385 stably binds to a key site in the inner face of the PR65 structure, stabilizing a conformation predisposed to associate with the catalytic and regulatory subunits of PP2A. DBK-766, in contrast, exhibits a weaker binding to the outer face of PR65 and elicits relatively large conformational fluctuations in PR65 when bound to the compact form.

## Graphical abstract

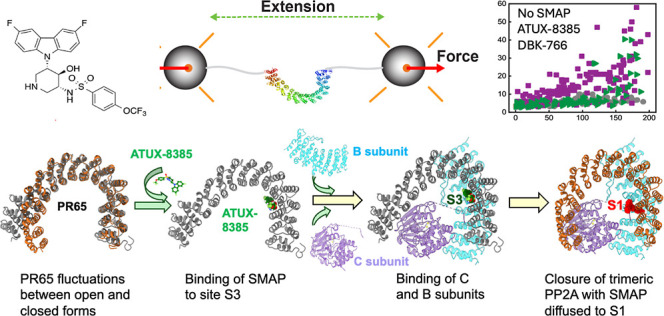

## Introduction

An intricate balance between kinase and phosphatase activities plays a vital role in signaling and protein homeostasis in the cell.^1,2^ Whereas many kinase inhibitors have been approved as treatments for human cancers, phosphatase inhibitors or activators have been less studied to date. Protein phosphatase 2A (PP2A), a serine/threonine phosphatase, belongs to a major class of enzymes regulating cell homeostasis by dephosphorylating key signaling molecules.^3^ Its dysregulation has been associated with diseases such as cancer, neurodegenerative disorders (Alzheimer’s and Parkinson’s), and cardiovascular and pulmonary diseases, making PP2A an attractive target for therapeutic interventions.^4,5^

PP2A is a heterotrimer composed of a scaffold (PR65 or subunit A), a catalytic subunit C, and a substrate-binding regulatory subunit B. A range of over 40 different B subunits, each specific for a distinct substrate or substrates, permits PP2A to control many different cellular signaling pathways. PR65 is a horseshoe-shaped tandem-repeat protein, composed of 15 HEAT (Huntingtin/Elongation/A-subunit/TOR1) repeats of 39 amino acids each, whose sequence similarity is relatively low.^2,6^ In the PP2A holoenzyme, the catalytic subunit binds to C-terminal repeats 11–15 of PR65, and the regulatory subunit binds to repeats 1–10.^3^ Crystal structures of different PP2A heterotrimers and of uncomplexed PR65 alone, as well as studies of their conformational dynamics,^7–10^ suggest that PR65 can adopt different conformations with varying degrees of compactness or extension and that PR65 needs to be highly flexible to accommodate the multitude of PP2A complexes necessary for diverse functionalities in the cell while maintaining its structural integrity. It has also been proposed in a landmark study that, rather than providing a rigid scaffold, PR65 acts as an elastic connector whose global fluctuations between open and closed forms modulate substrate binding and catalytic activity.^8^ Such coupling between global motions and catalytic activity has been pointed out to be common to many enzymes.^11^ The end-to-end distance of PR65 in different complexes differs by as much as 40 Å, indicating a remarkable scaffold flexibility^12^ amenable to modulation upon binding small molecules. Binding of small molecules could thus provide a mechanism for enhancing holoenzyme activity.

Certain classes of tricyclic sulfonamides (narcoleptics) have been shown to bind and activate PP2A, thereby acting as potential therapeutic molecules.^13–18^ These so-called SMAPs (small molecule activators of PP2A) include DT-061, which was shown to stabilize the heterotrimer upon binding to a pocket (Site 1, referred to subsequently as S1 or *S*_exp_) lined by all three PP2A subunits.^19^ This is the only resolved structure of PP2A complexed with a SMAP. The binding site of another SMAP, ATUX-8385, was mapped by hydroxyl footprinting experiments to PR65 residues K194-L198.^20^ Docking simulations near these residues showed that a site (S2), near repeats 4–6 of PR65, also presented a high-affinity pocket, but this pocket faced the exterior of the horseshoe-like fold of PR65, while S1 is lined by the inner helices of repeats 3–5 at the interface with the catalytic and regulatory subunits of PP2A.^10,21^ Extensive simulations further revealed a new site, S3, closely neighboring S1 that helped reconcile the experiments and computations. S3 has been proposed to serve as a first step for SMAP-binding PR65, succeeded by an induced diffusion to S1 upon complexation of PR65 with the catalytic and regulatory subunits of PP2A.^10^ ATUX-8385 and its enantiomer, ATUX-3364, have been studied for their effects on hepatoblastoma, a rare type of liver cancer. Both compounds effectively decreased the viability and proliferation of hepatoblastoma cells in vitro.^22^

In the present study, we explore the interaction of ATUX-8385 and DBK-766 (a nonfunctional SMAP) with PR65 using ensemble biophysical methods and examine their impact on protein folding using single-molecule optical tweezers.^23^ The two SMAPs have distinct effects on hepatoblastoma H-1650 cell lines: Whereas ATUX-8385 induces cell death,^24^ DBK-766 has no such effect. Dissection of the unfolding and refolding pathways of PR65 under force in the absence and presence of these small molecules reveals a global ‘chaperoning’ effect exerted by the functional SMAP on PR65, reminiscent of the effects of pharmacological chaperones (e.g., ref 25). In the presence of the nonfunctional SMAP, in contrast, PR65 exhibits smaller, more localized responses. We complement the experimental analysis by molecular dynamics (MD) simulations, repeated for the complexes of PR65 with ATUX-8385 and DBK766, both bound to site S3. ATUX-8385 binding stabilizes PR65 in a relatively extended form, which may help facilitate the insertion and binding of the C and B subunits prior to the subsequent tight assembly of the trimer into a compact form stabilized by ATUX-8385 bound to the interfacial site of the three PP2A subunits. In contrast, S3 does not accommodate stable DBK-766 binding, and instead, an alternative pose away from the trimer interface is selected. As such, DBK-766 would not promote/stabilize the binding of subunits B and C, which may explain its “nonproductive” nature.

## Results

### PR65 Binds Small Molecules ATUX-8385 and DBK-766

The relatively low solubility of the small molecules did not allow the use of biophysical methods, such as Isothermal Titration Calorimetry (ITC) to study binding to PR65. Consequently, alternative approaches were utilized. First, nanodifferential scanning fluorimetry (NanoDSF), a fluorescence-based label-free technique, was employed. NanoDSF measures changes in the intrinsic tryptophan and tyrosine fluorescence of proteins upon thermal denaturation. A shift in the protein melting temperature (*T*_m_) can indicate either stabilization or destabilization of the protein structure globally or locally, induced by ligand binding. Thermal denaturation of 2 μM PR65 was measured in 10% DMSO, in both the absence and presence of 100 μM ATUX-8385 and 100 μM DBK-766. An increase in the melting temperature of PR65 was observed with both ATUX-8385 and DBK-766 (from *T*_m_PR65_ = 52.7 ± 0.1 °C to *T*_m___PR65+ATUX_ = 53.5 ± 0.1 °C and *T*_m_PR65+DBK_ = 53.5 ± 0.1 °C), indicating binding and a small degree of stabilization of the protein structure (Figure 1a–c). The small shifts in *T*_m_ suggest dissociation constants in the micromolar range.

**Figure 1:**
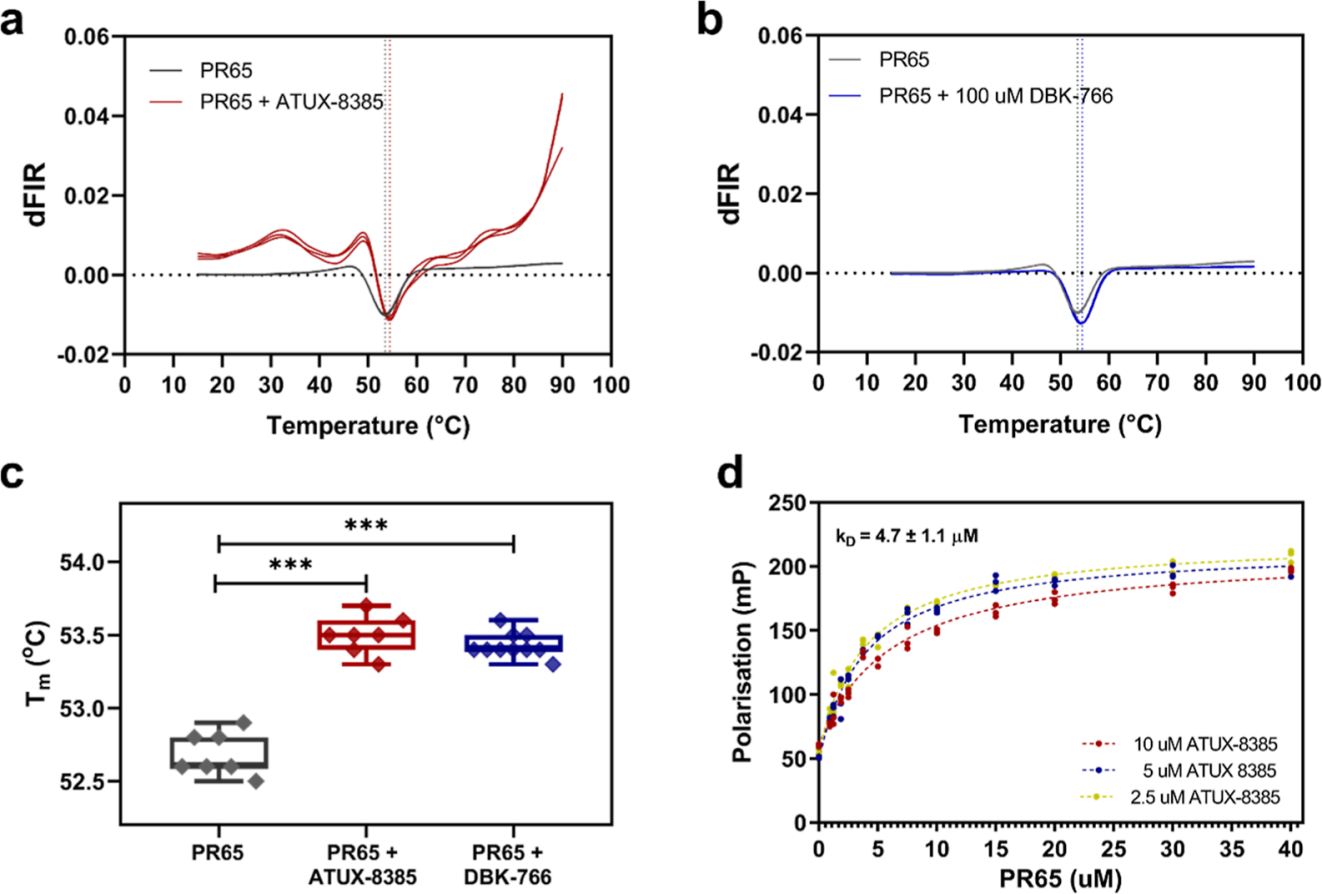
Small molecules ATUX-8385 and DBK-766 bind to PR65. (a) NanoDSF traces of the thermal denaturation of PR65 in the absence (gray traces) and in the presence of ATUX-8385 (red traces). The data shown are the first derivative of the ratio of fluorescence intensity read at 350 nm over that at 330 nm (dFIR (350 nm/330 nm)). The global minimum corresponds to the melting temperature of the protein, *T*_m_. A shift toward higher *T*_m_ values indicates an increase in stability induced upon SMAP binding (*T*_m_PR65_ = 52.7 ± 0.1 °C, *T*_m___PR65+ATUX_ = 53.5 ± 0.1 °C). (b) NanoDSF traces of the thermal denaturation of PR65 in the absence (gray traces) and presence (blue traces) of DBK-766. Again, *T*_m_PR65+DBK_ = 53.5 ± 0.1 °C. (c) Extracted *T*_m_ values from the NanoDSF traces indicate an upward shift in the presence of ATUX-8385 and DBK-766 (*p* < 0.001 via ordinary one-way ANOVA with multiple comparisons). The NanoDSF experiments were performed with a Prometheus NanoDSF instrument (NanoTemper Technologies), 2 μM PR65 in PBS, 2 mM DTT, incubated either with 10% DMSO, 100 μM ATUX-8385 or 100 M DBK-766 in a final concentration of 10% DMSO, and thermal denaturation was performed from 20 to 90 °C with a 1 °C/min rate. (d) Fluorescence polarization experiments show ATUX-8385 binding on PR65 with a dissociation constant in the low micromolar range. Shown are the fluorescence polarization values (mP) for 2.5 μM, 5 μM, and 10 μM ATUX-8385 upon PR65 titration, *N* = 3. One-site fitting of the data (see the Materials and Methods section) gives a *K*_D_ of 4.7 ± 1.1 μM.

To quantify the binding of ATUX-8385 to PR65, the fluorescence properties of ATUX-8385 were exploited. Upon excitation at 320 ± 10 nm, ATUX-8385 showed a maximum fluorescence intensity at 370 nm (Figure S1). Fluorescence polarization (FP) was employed to determine the dissociation constant. FP is based on the principle that the rotational motion of a fluorescent molecule affects the polarization of the emitted light. Upon binding to PR65, the rotational motion of ATUX-8385 was reduced, resulting in higher polarization values. PR65 was titrated into ATUX-8385 (10% DMSO) at 2.5 μM, 5 μM, and 10 μM (Figure 1d), which gave a dissociation constant (*K*_D_) of 4.7 ± 1.1 μM. DBK-766 is not fluorescent (Figure S1), precluding determination of its binding affinity by FP.

### Multiple (Un)­Folding Pathways of PR65 Detected by Optical Tweezers

Single PR65 molecules were tethered between polystyrene beads held in dual optical traps^26^ via 600 bp DNA handles (Figure 2a, see the Materials and Methods section).^27,28^ The constant velocity pulling experiments at 100 nm/s displayed heterogeneous unfolding/refolding behavior with hysteresis. Multiple intermediate states were observed during stretching curves with variations in extension, force, and the number of transitions in consecutive pulls and in different molecules (Figure 2b–e, red curves, red arrows, Figure S2a). The series of distinct unfolding transitions with varying extensions indicate that PR65 unfolds via domains of different sizes and stabilities due to varying cooperativity between the repeats and/or helix motifs.^29,30^ Similar heterogeneity was also observed during relaxation/refolding curves (Figure 2b,c, in blue, blue arrows). Interestingly, PR65 also displayed misfolding behavior,^31^ marked by stretching traces that showed intermediate states that could not be fully unfolded even when high forces were applied (Figure 2d–e, middle and right panels). Notably, these states were not observed in the first pulls (Figure S2b) and occurred abruptly in any successive stretching cycle (Figure 2d,e), suggesting that the behavior was due to the incorrect folding of some subdomains that disrupted the folding of the entire protein.

**Figure 2:**
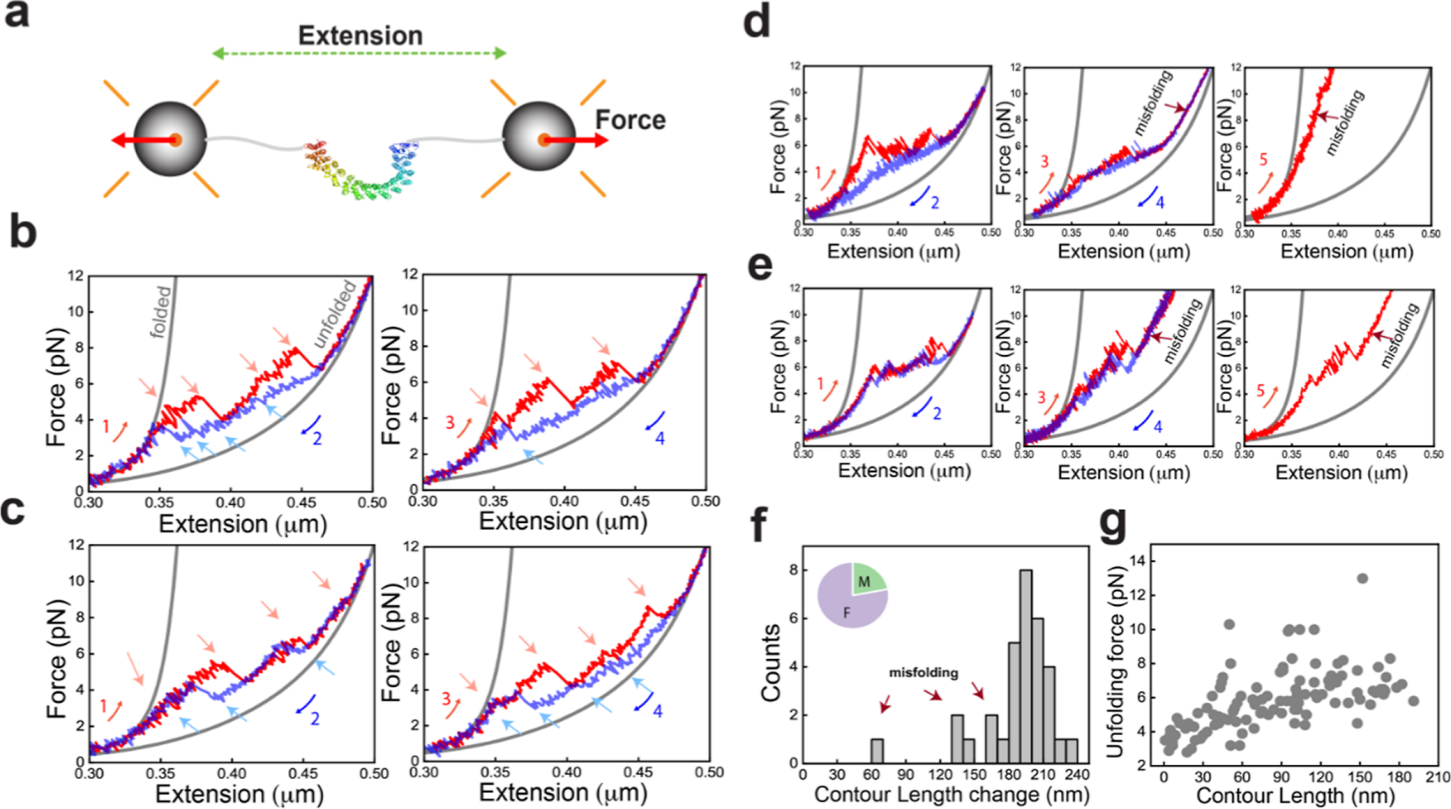
Single molecule (un)­folding of PR65. (a) Mechanical manipulation of single PR65 molecules using a dual-beam optical tweezers setup. (b) Stretch (red)–relax (blue) cycles showing multiple intermediate states and heterogeneity in the lengths of unfolding (red arrows)/refolding (blue arrows) rips and number of intermediates in successive cycles. Numbers denote the order of the pull–relax cycles. Gray curves are WLC fitting. (c) Example force extension traces of another PR65 molecule showing variability in the stretching and relaxation curves compared to the molecule in b. (d,e) Example force extension traces for molecules showing abrupt misfolding events in subsequent stretching curves after the first pull–relax cycle. (f) Contour length change (Δ*L*_c_) distribution from the folded to unfolded state (b) from WLC fitting of stretching curves (*N*_cycles_ = 32, *N*_molecules_ = 16). The inset shows the percentage of stretching curves that displayed misfolding (M) and full folding (F). (g) Unfolding force vs absolute contour length (*L*_c_) of each intermediate state observed during pulling (*N*_cycles_ = 32, *N*_molecules_ = 16).

We next fitted the folded and unfolded branches (Figure 2b, gray curves) of the force–extension curves (FECs) to the extensible Worm-like chain (WLC) model^32^ (Methods). The distribution of the contour length change of unfolding (Δ*L*_c_) displayed a major peak at 198 ± 1.4 nm matching the expected length of 200 nm (Figure 2f). The average force for complete unfolding of PR65 was determined to be ∼7.2 pN at 100 nm/s pulling speed. The shorter lengths in the distribution correspond to the misfolded fraction (22% of the total stretching traces) that could not be fully unfolded, even when high forces were applied (Figure 2f inset).

We quantified the force and absolute contour length (*L*_c_) prior to each unfolding and refolding rip (Figure 2g; see the Materials and Methods section) from those traces that showed no misfolding. The absence of clustering of data points indicates a population of multiple intermediate states and a complex network of interactions between the helix-motifs. These intricate interactions result in distinct unfolding transitions of domains with varying stabilities in each stretch–relax cycle. The heterogeneity in the unfolding/refolding forces and contour lengths of transitions in our single-molecule experiments is highly reminiscent of the behavior observed for chemical-induced unfolding of PR65 in ensemble measurements and shown to arise from multiple pathways.^30^ The presence of multiple pathways of similar energy for (un)­folding has been reported by our group and others for several repeat proteins^33–38^ including PR65. This feature reflects the high internal symmetry and is in striking contrast to globular proteins for which there are generally only single pathways accessible (for both chemical- and force-induced unfolding).^39,40^

### ATUX-8385 Binding Globally Stabilizes PR65 and Prevents Misfolding

To understand how functional SMAPs interact with PR65, we repeated the optical tweezers experiments at 100 nm/s in the presence of ATUX-8385 (Figures 3 and S3). Interestingly, most PR65 molecules displayed higher unfolding forces than those in the absence of ATUX-8385 for each transition during stretching (compare Figures 2b,c and 3b–d), and importantly, higher resistance to deformation was observed starting from the first pulls, indicating an increase in the stability of the folded state in the presence of the functional SMAP (Figure 3b,c, red curve and Figure S3b). A subset of molecules showed stabilization of only a few subdomains of PR65, indicating a different binding mode (Figure 3d). Similar increases in the refolding forces were also observed during relaxation, with variation in the hysteresis between stretch–relax cycles (Figures 3b,c,g and S3c). The heterogeneity in the extensions, forces, and number of transitions within successive stretch–relax cycles and between different molecules was similar in the presence and absence of SMAP (Figures S2a and S3a). Notably, and in striking contrast to the no-SMAP data, no misfolding was observed in the presence of SMAP, with Δ*L*_c_ values showing a single sharp peak at 196 ± 3 nm, indicating high population of the folded state (Figure 3e).

**Figure 3:**
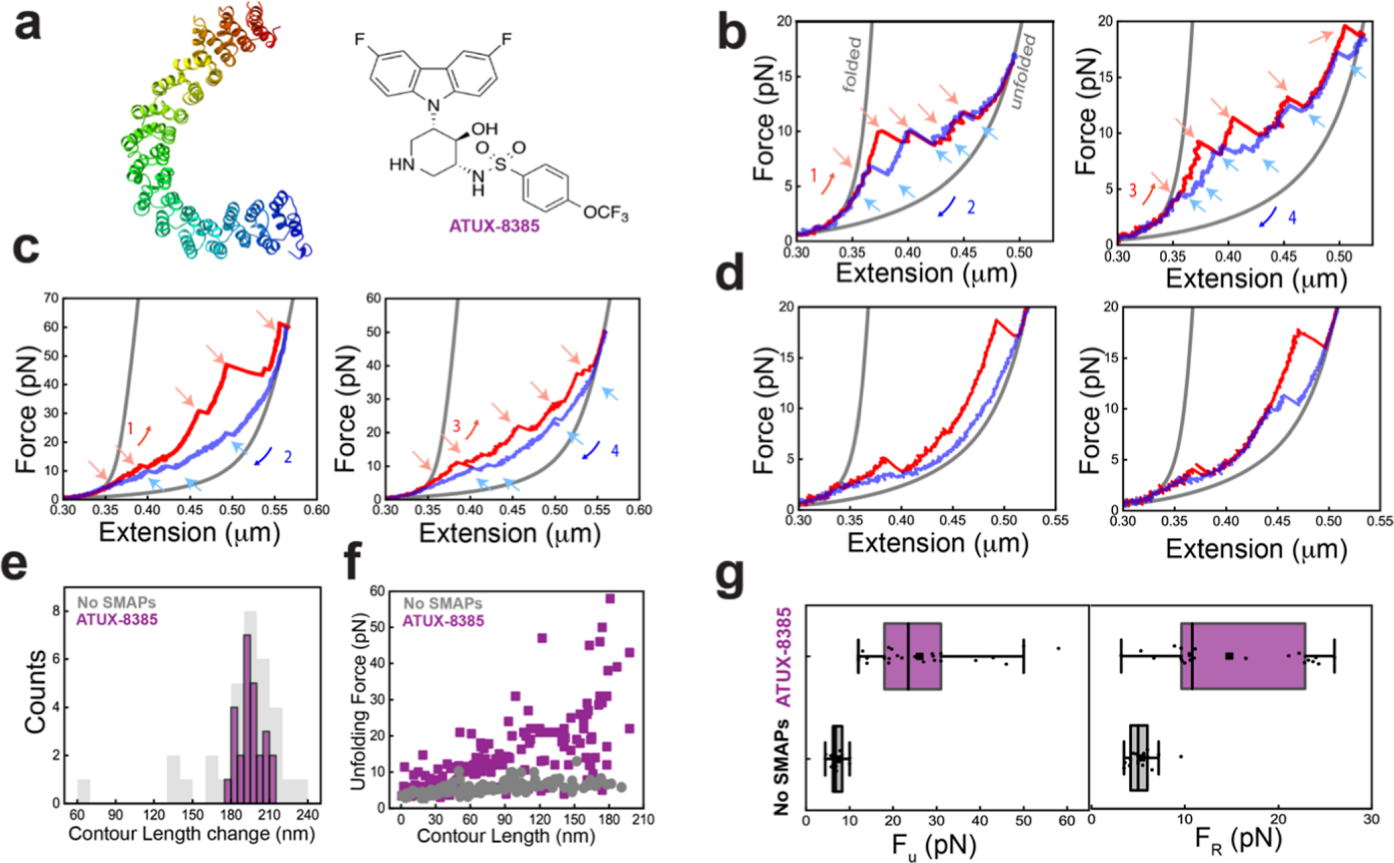
ATUX-8385 acts as a chaperone in the folding of PR65. (a) Structure of the tricyclic neuroleptic compound ATUX-8385. (b) Force–extension curves of PR65 from pull–relax experiments at 100 nm/s, in the presence of 10 μM SMAP dissolved in <2% DMSO in Tris–HCl buffer (see further details in the Materials and Methods section). Stretching (red) and relaxation (blue) curves from two consecutive cycles showing high unfolding and refolding forces of each rip. Gray curves are WLC fitting. (c) Example force–extension traces from consecutive cycles from another PR65 molecule displaying heterogeneity in the unfolding and refolding pathways with larger hysteresis (left panel) compared to the molecule in (b). (d) Example force–extension traces from two separate PR65 molecules (panels right and left) showing stabilization of a few domains of PR65. (e) Δ*L*_c_ distribution quantified (in purple) from all the stretching curves (*N*_cycles_ = 26, *N*_molecules_ = 16) in the presence of ATUX-8385, showing a single peak corresponding to the folded PR65, in contrast to the misfolding fractions observed for the no-SMAP condition (in gray). (f) Unfolding force versus absolute contour length (*L*_c_) of each intermediate state (purple with SMAP, gray without SMAP (from Figure 2g)) observed during pulling (*N*_cycles_ = 26, *N*_molecules_ = 16). Data showing ATUX-8385 binding stabilizes the entire PR65 folded state. (g) Box plots showing distribution of maximum unfolding force (left panel) and refolding force (right panel) with (*N* = 26, 24, purple) and without (*N* = 23, 19, gray) SMAP.

The large difference in the plots of unfolding force versus L_c_ in the presence versus the absence of SMAP (Figure 3f and left panel of Figure 3g) suggests that SMAP binding globally stabilizes the folded state of PR65. This effect could arise from SMAP binding at multiple sites or binding at a single binding site having a long-range effect on the folded structure, increasing the thermodynamic stability and the barrier to unfolding. The interactions of SMAP with PR65 also prevent its misfolding.

## Binding of Nonfunctional SMAP, DBK-766, Impedes the Folding of PR65

When the unfolding experiments were performed in the presence of the nonfunctional SMAP, DBK-766, the data for the first pulls show that there are higher unfolding forces compared with the no-SMAP condition, but for only a few repeats of PR65 (Figure 4a,b, left panels, Figure 4c). This behavior was also observed in the small subset of traces in the presence of ATUX-8385 (Figure 3d), indicating a limited mode of interactions in those cases. Interestingly, however, in subsequent stretch–relaxation cycles, no significant unfolding/refolding transitions were observed (Figure 4a,b right panels), and the PR65 molecule appeared to be locked in the unfolded state for multiple cycles before breaking. Since this locking behavior was never observed for either ATUX-8385 or no-SMAP conditions, it suggests a distinct mode of interaction of DBK-776 with the unfolded state of PR65. The Δ*L*_c_ distribution (Figure 4d) thus showed two distinct peaks for the folded and unfolded lengths (33% of the traces). DBK-766 has a significantly different mode of interaction with both the folded and unfolded states of PR65, as compared with the functional ATUX-8385 SMAP (Figure 4c). These data suggest that DBK-766 binds weakly to the folded state but significantly stabilizes the unfolded state of PR65, hindering the folding of the protein.

**Figure 4:**
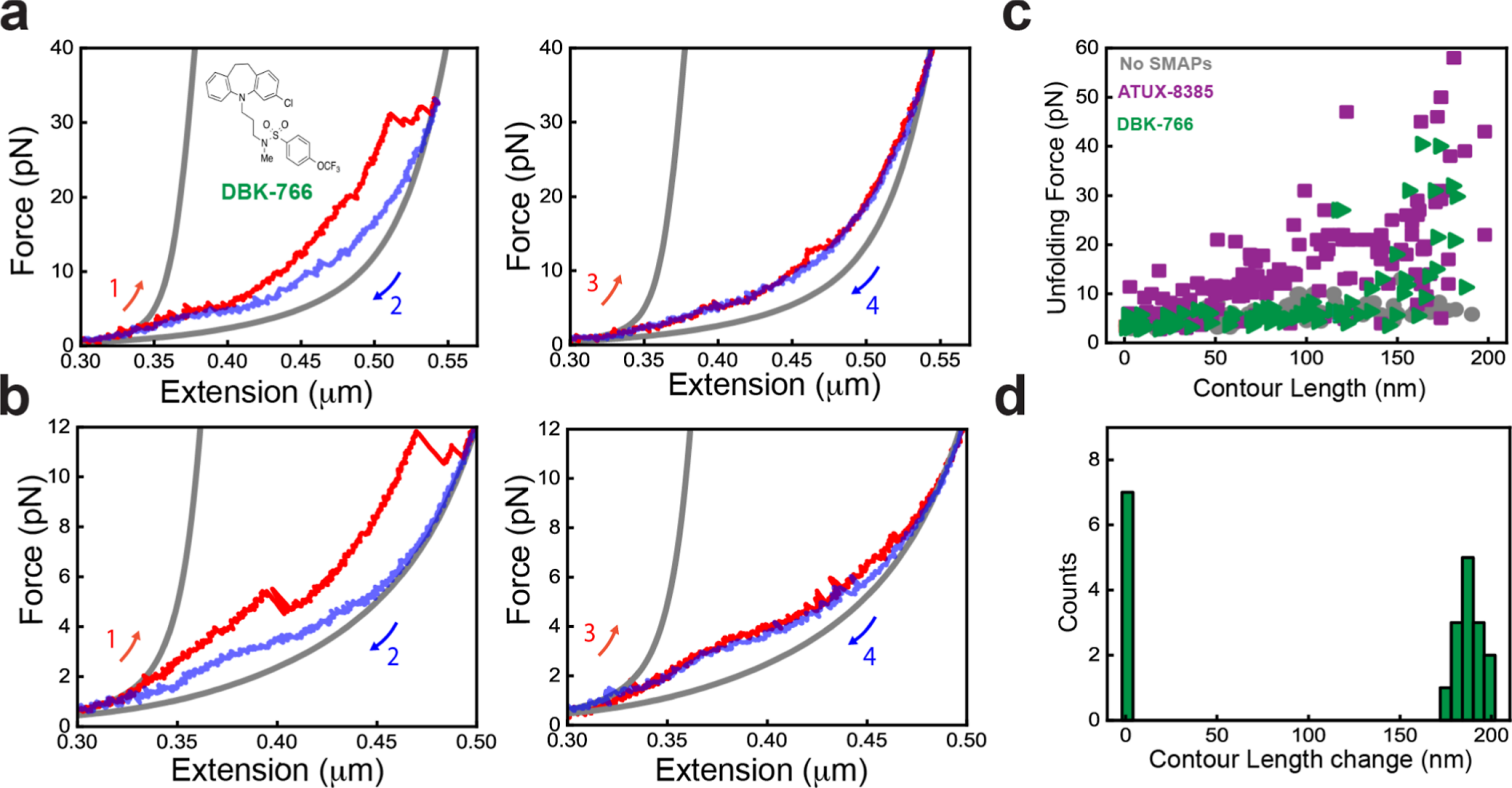
DBK-766 binding impedes the folding of PR65. (a,b) Force extension curves of PR65 pull–relax experiments at 100 nm/s, in the presence of 10 μM DBK-766 SMAP (inset) dissolved in <2% DMSO in Tris–HCl buffer (see the Materials and Methods section). Stretching (red) curve (left panel) showing high unfolding force of a few repeats, while no significant refolding jumps were observed during relaxation (blue). In the next pull–relax cycle (right panel), the molecule remained unfolded. Gray curves are WLC fitting. (c) Unfolding force vs absolute contour length (*L*_c_) of each intermediate state observed during pulling (with DBK-766 in green (*N*_cycles_ = 21, *N*_molecules_ = 10), with ATUX-8385 in purple (from Figure 3f), and without SMAP in gray (from Figure 2g)). Data showing weak stabilization by DBK-766 binding as compared to ATUX-8385 binding. (d) Δ*L*_c_ distribution quantified from all the stretching curves (*N*_cycles_ = 21, *N*_molecules_ = 10) in the presence of DBK-766, showing one peak corresponding to the folded PR65 and another peak at 0 nm corresponding to the pulls, showing no significant unfolding transitions (Figure 4a,b right panels).

### Binding Site S3, in Proximity of Site S1 Resolved for DT-061, Shows High Affinity for ATUX-8385 but Not for DBK-766

Although both ATUX-8385 and DBK-766 are tricyclic sulfonamides, the results presented above show that they exhibit different responses to uniaxial tension. We undertook a deeper examination of the potential binding properties of these two SMAPs on PR65 for a better understanding of the molecular basis of their distinctive effects in the force–extension experiments.^19^ Previous label-free single-molecule experiments with nanoaperture optical tweezers combined with MD simulations gave first insights into the effects of ATUX-8385 binding on PR65 conformational behavior and its optical scattering properties,^21^ followed by recent extensive simulations of the binding properties of ATUX-8385 and equilibrium dynamics of PR65 bound to ATUX-8385.^10^ However, no (experimental or computational) study of the characterization of the binding properties of DBK-766 has been conducted to date. Here, we present a comparative study.

First, we present the results for ATUX-8385 bound to site S3. Figures 5a,b and S4a display the binding site and pose from different perspectives. As mentioned above, this site has been previously identified to be a high-affinity site for ATUX-8385, and the SMAP may resettle via induced rearrangements in the adjacent site S1 that has been experimentally observed to bind another productive SMAP DT-061 in the PP2A trimer. Figure 5a shows the two overlaid conformations of PR65, extended (light green, adopted as a starting conformer in simulating ATUX-8385 (red, space-filling) binding) and compact (dark green, resolved by cryo-EM for the trimer complexed with DT-061) in the presence of ATUX-8385 bound to S3. The binding pose of DT-061 (orange sticks) is also shown to illustrate its proximity to S3.

**Figure 5:**
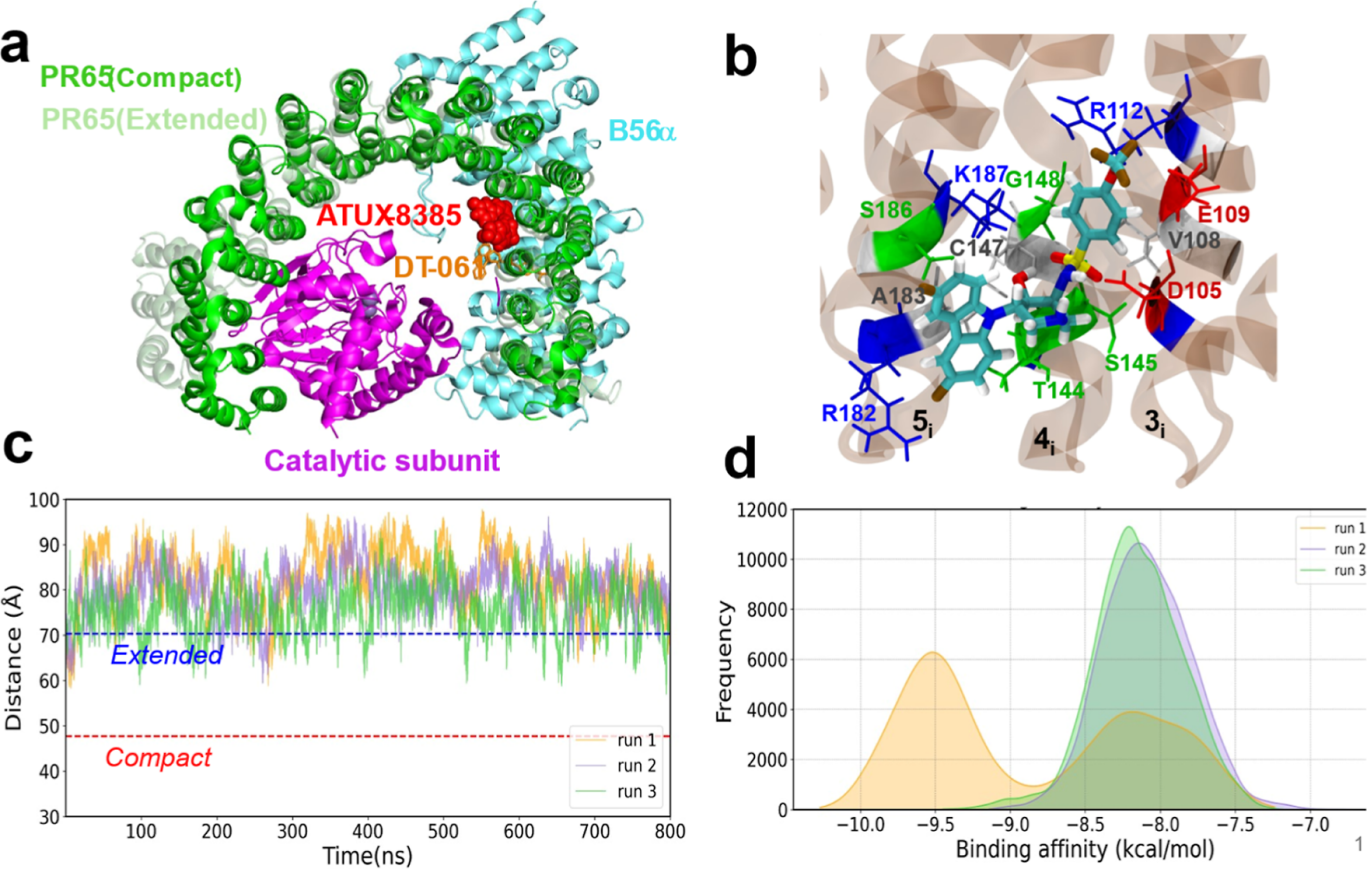
Results from docking and molecular simulations of ATUX-8385 binding to PR65. (a) Binding site S3 of ATUX-8385 to PR65 (predicted by docking simulations and confirmed by MD simulations), adjacent to the position of SMAP DT-061 (resolved by cryo-EM). Both extended (apo, light green; based on PDB: 1B3U^6^) and compact (green, bound to catalytic (magenta) and regulatory (cyan) subunits) are shown. (b) Close-up view of ATUX-8385 at site S3 in the complex with PR65. ATUX-8385 is shown in teal licorice representation. The coordinating residues of PR65 from the inner helices of repeats 3–5 (designated as 3_i_, 4_i_, and 5_i_) are shown in sticks and labeled. PR65 is rendered with a transparent brown background. (c) Time evolution of PR65 end-to-end distance in the presence of ATUX-8385, based on N29–F577 α carbons (see Figure S4a). Three independent MD runs of 800 ns each were performed for the extended form of PR65. ATUX-8385 maintains its original binding pose (except for a reorientation within S3 in run 1 (see Figure S4d)). PR65 maintains its extended conformation in all three runs. The dashed horizontal lines indicate the end-to-end distances corresponding to the experimentally resolved open/extended and closed/compact states. (d) Distribution of binding energies observed in MD runs. Binding affinities were evaluated using PRODIGY-LIG. Histograms (and traces) corresponding to runs 1, 2, and 3 are colored light orange, purple, and green, respectively, in panels c and d.

MD simulations (in triplicate, 800 ns each) showed that ATUX-8385 remained bound to S3 and contributed to the stabilization of the PR65 extended form, as can be seen from the time evolution of the PR65 end-to-end distance (Figure 5c) and the histogram in Figure S4b. Figure S4c illustrates how contacts between ATUX-8385 and PR65 key residues (D105, R182, A183, S186, and K187) were maintained at site S3 during the entire duration of the three independent runs. Interestingly, ATUX-8385 maintained its original pose at S3 in two of the runs (with a root-mean-square deviation (RMSD) of <5 Å with respect to the starting pose), whereas its RMSD increased to ∼8.5 Å at around 350 ns in the other run (run 1; Figure S4d). The sudden hike in RMSD originated from the flipping of ATUX-8385 within the same site S3 to find an even more favorable pose. The histogram of its the binding affinity of ATUX-8385 shows two peaks, at −8.2 kcal/mol and −9.5 kcal/mol (Figure 5d), the latter corresponding to the flipped orientation. Binding affinities were calculated using PRODIGY-LIG^41^ for (8,000) snapshots collected at 100 ps intervals in each run.

Similar simulations performed with DBK-766 revealed a different behavior. Binding of DBK-766 to S3 was substantially weaker, and MD simulations led to its dissociation in all three runs, while the end-to-end distance showed broad fluctuations (Figure 6a). The instability of DBK-766 is illustrated by the broad distribution of its RMSD histogram in Figure 6a, compared to that of ATUX-8385. In all three MD runs (of 800 ns each), the DBK-766 RMSD with respect to its original pose exceeded 25 Å, consistent with its dislocation from S3 and complete dissociation. Figure 6c showcases one such instance (run 1), where dislocation of DBK-766 from site S3 is observed at 300 ns, followed by further movement and complete dissociation at 333 ns during MD run1.

**Figure 6:**
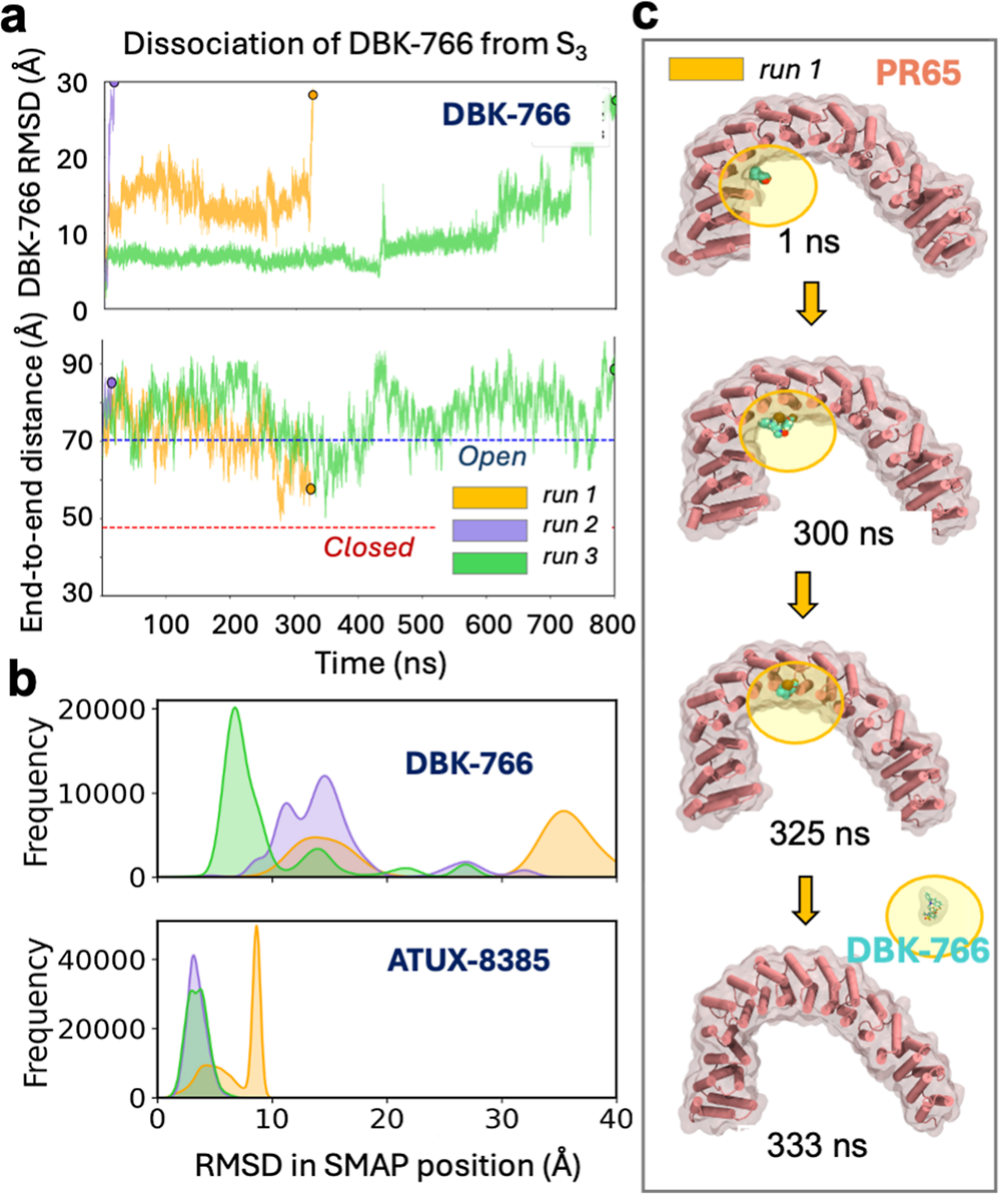
DBK-766 is not stable at site S3. (a) Results from three independent runs (same simulation conditions as in Figure 5 except for replacement of ATUX-8385 by the optimal binding pose of DDK-766 at S3) show that DBK-766 cannot stably bind to S3, and it dissociates at t ≈ 330, 25, and 790 ns in the three respective runs. The lower panel displays the fluctuations in PR65 end-to-end distance prior to its dissociation. (b) RMSD histograms of DBK-766 with respect to the initial pose in runs 1–3 (top) are significantly higher than those of ATUX-8385 (bottom), which remains bound to S3 in all runs. (c) Four snapshots illustrating the time evolution of DBK-766—PR65 interactions in run 1. The DBK-766 instantaneous pose is highlighted to indicate its gradual dislocation and complete dissociation.

### DBK-766 Preferentially Binds an Alternative Site that Does Not Allow for Engaging in Ternary Interactions that Promote the Assembly of PP2A Subunits

DBK-766 is a nonfunctional SMAP, but experimental evidence indicates that it is capable of binding to PR65, suggesting the existence of an alternative stable binding site that does not lend itself to PP2A holoenzyme formation/activation. To explore whether an alternative site exists, we carried out additional docking simulations and identified a novel binding site on the outer (convex) surface of HEAT repeats 7 and 8 (7_o_ and 8_o_) (Figure 7a). MD runs conducted in triplicate starting from DBK-766 bound to that site using the open conformation of PR65 showed that DBK-766 remained stably bound in two runs (run 2 and run 3), while it dissociated in run 1. Its binding affinity in these two runs was comparable to that of ATUX-8385 binding to S3 (Figure S5). The SMAP RMSD profiles and histograms (Figure 7b,c top panels) indicated the high stability of DBK-766 in the two runs (in sharp contrast to Figure 6a,b top). DBK-766-bound PR65 maintained its extended end-to-end distance (Figure 7b,c bottom panels). Yet, the dissociation observed in one of the runs signaled that DBK-766 binding strength was relatively weaker than that observed for ATUX-8385 at site S3.

**Figure 7:**
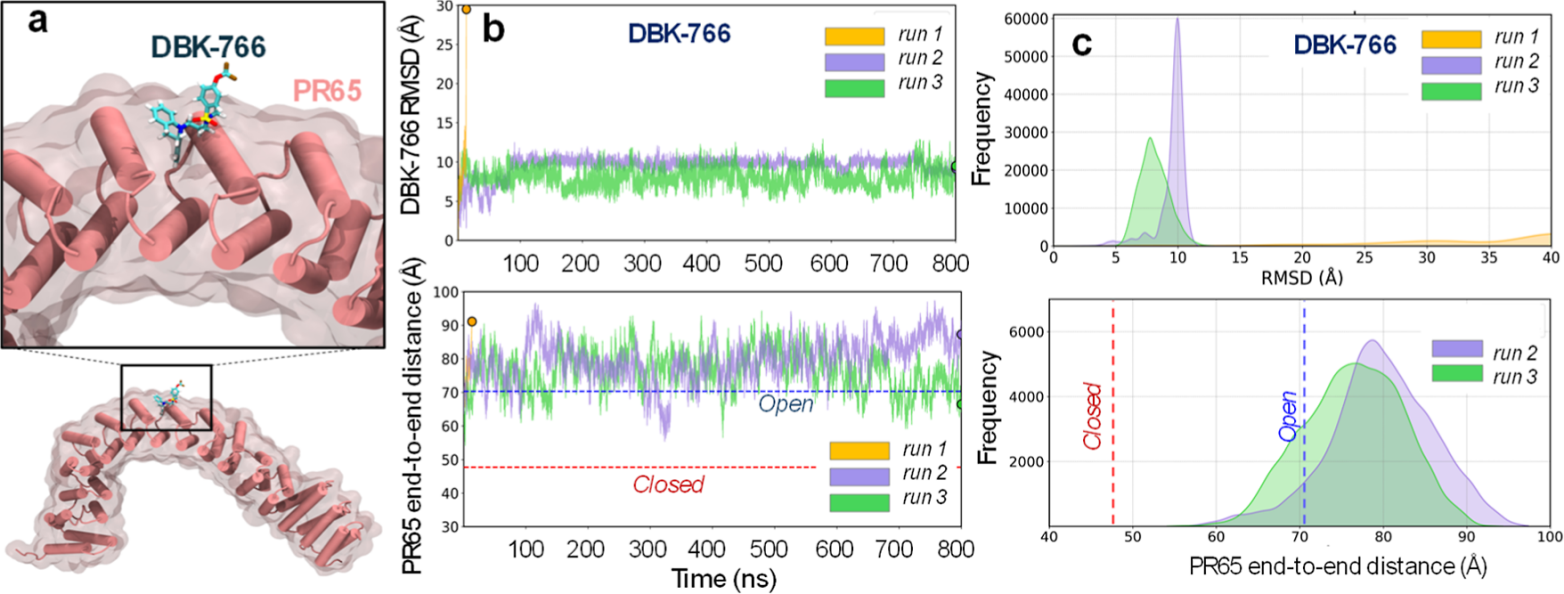
DBK-766 binding at an outward-facing site of PR65, coordinated by the outer helices of repeats 7 and 8. (a) DBK-766-bound PR65 predicted by computational docking simulations and verified by MD simulations to be a stable pose in two of the three runs initiated with the bound form. (b) Top panel: RMSD fluctuation of DBK-766 calculated from MD snapshots shows stable binding in two runs (green and purple) but dissociation in the third (orange). Bottom panel: Time evolution of PR65 end-to-end distance obtained from MD simulations. (c) Top panel: RMSD distribution of DBK-766 binding to an outward-facing site. Bottom panel: distribution of PR65’s end-to-end distance deduced from MD snapshots. Results are shown for the two runs that maintained DBK-766 in the bound state.

As a further test, we examined whether the propensity of DBK-766 to bind this relatively favorable site depended on the PR65 conformation, i.e., whether it would stably bind to the closed form of PR65, which might open a cavity to better accommodate its binding. Simulations indeed showed DBK-766 remained stably bound and underwent only small rearrangements within the confines of this site (Figure S6a,c). Notably, PR65 exhibited quite large fluctuations during those simulations. The large (40 to 90 Å) and rapid changes in the PR65 end-to-end distance (Figure S6b and d) point to a possible destabilizing effect by DBK-766, consistent with the potential dissolution of structure observed in experiments. This is in contrast to the stabilizing effect typical of the SMAPs.

In summary, these computations show that DBK-766 does not bind to S3 and instead prefers binding to an outward-facing site, and binding to that site is stronger when PR65 is in the closed state. In contrast to productive SMAPs, DBK-766 does not stabilize PR65; instead, PR65 exhibits a large size and frequent transitions between open and closed states (the end-to-end distance even exceeding in both directions those observed in resolved structures in the respective open and closed states). Finally, this site lies on the outer face of PR65, and as such, it would not be expected to assist in strengthening the interactions with the other two subunits or enhancing PP2A catalytic activity.

## Discussion

Repeat-protein folding has been widely studied,^33–36,42–44^ and the behavior has been shown to be distinct from that of globular proteins, reflecting the linear, repetitive, and nonglobular architecture. Our recent elastic network analysis of a series of tandem repeat proteins also pointed to their unique ability of being highly stable (to serve as a scaffold) and flexible (to adapt to, if not facilitate, conformational changes involved in functional events) at the same time.^45^ The potential sensitivity of the substrate binding and catalytic activity of PP2A to mechanical force was first pointed out by Karplus, Kleckner, and co-workers, who noted that the soft modes intrinsically accessible to PR65 could open/close the substrate-binding or active site interface.^8^ A more recent study of PP2A dynamics by our groups further showed how the fluctuations between open and closed forms of PR65 indeed help in opening/closing the catalytic cleft between the domains 1 and 2 of the catalytic subunit C, assisted by the regulatory B subunit.^7^ Despite these insights gained from computations, the mechanics of repeats proteins, and of PR65 in particular, have been much less well-studied experimentally than those of globular proteins as helical-repeat proteins like PR65 tend to easily deform and require an optical tweezer to access the low associated forces, which are below the limit of atomic force microscopy.^46–49^ Here, we probed the mechanical properties of the PR65 nanospring and the effects of binding of small molecule activators of PP2A (SMAPs) to help fill this knowledge gap.

Our observations demonstrate how mechanical forces and SMAP binding may affect not only the substrate binding or catalytic activity of PP2A but also the unfolding/refolding behavior of PR65, depending on the type of SMAP.^13–18^ We find that PR65 unfolding under force is very heterogeneous, which is reminiscent of the chemical-induced unfolding pathways of repeat proteins, including PR65, arising due to their structural symmetry proteins.^33–38^ Strikingly, ATUX-8385 binding has a global stabilizing effect on PR65 and prevents misfolding events. This behavior is similar to that of molecular chaperones such as the heat-shock proteins and has been mimicked by small-molecule “pharmacological” chaperones^25,50,51^ that have been developed as drugs to prevent proteins from unfolding and misfolding and restore stability to destabilizing mutations associated with diseases such as cancer, cystic fibrosis, and lysosomal storage disorders by stabilizing their native states.

For PR65, which we have shown previously to populate partly unfolded states at physiological temperature,^30^ our results raise the possibility that the (productive/functional) SMAP helps prevent misfolding events and guide its correct refolding. In contrast to ATUX-8385, the nonfunctional SMAP DBK-766 has a very different mode of interaction, and following the first response to stretch–relax cycle (which indicates binding, albeit weaker than that of ATUX-8385), it appears to lack local structure formation/dissolution steps and instead exhibits a smooth response indicative of an unfolded/disordered state and the absence of PR65 refolding. Given its dramatically toxic effect on PR65 refolding, further exploration of DBK-766 in cells would be warranted to see if there are any deleterious effects on PP2A function (rather than no effect). Interestingly in this regard, some disease conditions (e.g., diabetes^52^) result from hyperactivation of PP2A, and it would be interesting to explore the effects of the SMAPs, particularly DBK-766, in such a context.

Toward understanding the molecular basis of the distinctive mechanical behaviors of ATUX-8385-bound and DBK-766-bound PR65, we performed comparative simulations of the binding properties of these two SMAPs and the conformational dynamics of PR65 bound to either SMAP. In our recent work, site S3 emerged as a major site for ATUX-8385 binding, but the behavior of DBK-766 was unknown. S3 was distinguished by its close proximity to the site S1 resolved for DT-061; it shared coordinating residues such as D105, suggesting that it might serve as an intermediate site prior to settling to S1 by an induced fit upon PR65 trimerization. Current extensive simulations also showed the adaptability of ATUX-8385 bound to S3 to optimize its binding pose within the pocket (including a conformational flip to achieve tighter binding), noted in Figures 5 and S4, which further supported the predisposition of this site to induced fit, as well as to the contribution of entropic effects to the selection of S3 by ATUX-8385. Furthermore, the computed binding affinity of ATUX-8385 to S3 was comparable to the experimental *K*_D_ values. In contrast, DBK-766 did not stably bind to S3 (Figure 6). An alternative site identified on the exterior face of the repeat units (Figure 7) could bind DBK-766, preferably in the compact state of PR65. Yet, binding to the compact form gave rise to pronounced fluctuations in the PR65 end-to-end distance, indicating a possible destabilizing effect; and binding to the extended form, though less destabilizing, was relatively weaker as one of the three runs resulted in SMAP dissociation. Even though binding to this site favored an open conformation, it would have no effect on stabilizing the association of PR65 with B and C subunits (because they bind to the interior face of PR65).

As a final test, we examined whether S3 would be a preferred binding site on PR65 (alone) for DT-061 as well, which would support our hypothesis that S3 serves as a first binding site on PR65 prior to its assembly with the other two subunits. Blind docking simulations unambiguously demonstrated that site S3 was selected as the highest-affinity site by DT-061 (Figure S7), in support of the significance of site S3 for (functional) SMAP binding to PR65, before trimerization. Overall, the simulations suggest a 3-step mechanism preceding the SMAP-enhanced activation of PP2A: (1) binding of SMAP to the open form of PR65, at site S1; (ii) insertions of subunits C and B, and (iii) overall compaction of the trimeric structure accompanied by the induced diffusion of SMAP toward S1 to enable optimal interactions across the three subunits of the phosphatase.

In summary, the optical tweezers analysis presented here reveals a global stabilizing effect of functional SMAP ATUX-8385, which prevents misfolding of PR65 and contrasts with the nonfunctional SMAP DBK-766, which acts instead to impair PR65 refolding. The simulations provide atomistic insights into the distinct binding modes of the SMAPs (with the caveat that confirmation of the multiple binding poses would require experimental structural verification): the functional SMAP ATUX-8385 facilitates the holoenzyme activity by predisposing the PR65 structure to complexation with subunits B and C and by consolidating its intrinsic dynamics as described schematically in Figure 8; however, the nonfunctional DBK-766 preferentially binds another site, with either weaker affinity or the potential to disrupt the conformational dynamics of PR65 and thereby the cooperativity required for PR65 function.

**Figure 8:**
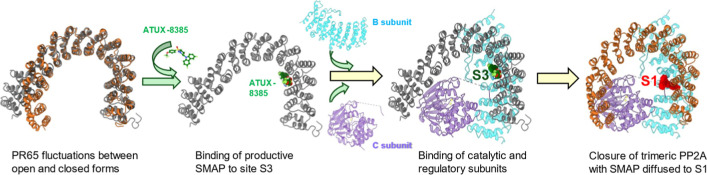
Proposed mechanism of binding of SMAP to PR65 and subsequent PP2A activation. The SMAP (here, ATUX-8385) first binds to site S3 to promote PR65 opening to facilitate the binding of the catalytic and regulatory subunits to the scaffold PR65, followed by overall compaction and sliding of the SMAP to site S1 by induced fit.

## Materials and Methods

### Expression and Purification of PR65

The expression and purification of WT and ybbr-tagged PR65 were performed as previously described.^30^ In brief, the plasmid encoding PR65 was transformed into chemically competent C41 *E. coli* (Komander laboratory, MRC-LMB, Cambridge). Cultures were grown at 37 °C in 2xYT medium containing ampicillin (50 μg/mL) until an OD_600_ of 0.6 to 0.8 was reached. Protein expression was induced with 500 μM isopropyl-β-d-thiogalactopyranoside (IPTG) (Generon) at 25 °C overnight. Cells were harvested by centrifugation at 4000*g* for 10 min at 4 °C before being resuspended in lysis buffer (50 mM Tris–HCl pH 7.5, 500 mM NaCl, 2 mM dithiothreitol (DTT)) supplemented with EDTA-free protease inhibitor cocktail (Sigma-Aldrich) and deoxyribonuclease (DNase) I (Sigma-Aldrich). The cells were lysed by passing the suspension two to three times through an Emulsiflex-C5 (AVESTIN) at pressures of 10,000 to 15,000 psi. Soluble protein was separated from cell debris and other insoluble fractions by centrifugation at 35,000g for 35 min at 4 °C. The soluble protein fraction was applied to glutathione resin (Amintra Affinity, EGTA (0.5 g/L), 2 mM DTT), the GST tag was cleaved with thrombin, and PR65 was eluted using a gravity column. After the column was washed, the protein was subsequently eluted using a 20× column-volume salt gradient from 0 to 1 M NaCl. MonoQ fractions containing the protein were concentrated before application to a HiLoad 26/600 Superdex 200 pg (GE Healthcare) equilibrated in phosphate-buffered saline (pH 7.4) and 2 mM DTT. Samples were analyzed by SDS-PAGE (polyacrylamide gel electrophoresis) by comparing the lysed, flowthrough, and eluted fractions. The identity of the protein was confirmed via MALDI mass spectrometry (Department of Chemistry, University of Cambridge).

### Nano-Differential Scanning Calorimetry

PR65 samples (2 μM) in PBS and 2 mM DTT were mixed with ATUX-8385, or with DBK-766, to a final concentration of 100 μM, 10% DMSO in a total volume of 20 μL. The mixtures were allowed to equilibrate at room temperature for 10 min. High-sensitivity capillaries (NanoTemper) were filled with the equilibrated PR65-SMAP mixtures by using capillary forces, ensuring no air bubbles were present. The capillaries were loaded into the Prometheus NT.48, and a thermal ramp from 20 to 90 °C at a rate of 1 °C per minute was applied. Intrinsic tryptophan and tyrosine fluorescence at 330 and 350 nm were continuously monitored. The melting temperature (*T*_m_) was determined from the first derivative of the fluorescence ratio (I_350_/I_330_) with respect to temperature. The *T*_m_ corresponds to the inflection point of the melting curve.

### Fluorescence Polarization

A fixed concentration of ATUX-8385 (2.5 μM, 5 μM, and 10 μM) was mixed with varying concentrations of PR65 (0 to 80 μM), in PBS +2 mM DTT, to a total volume of 20 μL in each well of a 384-well microplate (Greiner) and to a final DMSO concentration of 10%. The plate was incubated at 40 °C for 30 min to ensure binding equilibrium. Fluorescence polarization was measured at 40 °C using an excitation wavelength of 295 ± 10 nm and an emission wavelength of 360 ± 20 nm on a CLARIOStar plate reader (BMG Labtech), measuring the light in parallel and perpendicular planes relative to the excitation plane. Wells containing only PR65 were used as controls to determine background fluorescence. Fluorescence polarization (P) was calculated using the equation

1 $$P=\frac{I_{∥}-I_{⊥}}{I_{∥}+I_{⊥}}$$

where *I*_∥_ and *I*_⊥_ are the intensities of fluorescence parallel and perpendicular to the excitation plane, respectively.

Nonlinear regression analysis was performed using a one-site binding model to fit the data and determine the dissociation constant *k*_D_, using the following equation

2 $$P=\frac{P_{\max}\times C_{\mathrm{PR}65}}{k_{D}+C_{\mathrm{PR}65}}$$

where *P* is the polarization, *P*_max_ is the maximum polarization at the plateau, *C*_PR65_ is the concentration of PR65 (μM), and *k*_D_ is the equilibrium dissociation constant.

### Protein–Ligand Docking Simulations

Docking simulations were performed using AutoDock Vina.^53^ In preparation for docking simulations, hydrogen was added to both the receptors and the SMAP, and the resulting structures were saved in the PDBQT file format compatible with Vina, utilizing AutoDock Tools1.5.6.^54^ Subsequently, partial charges for SMAP were calculated by using the Gasteiger method. Simulations were repeated with a series of three-dimensional cubical boxes, with edge lengths ranging from 20 to 80 Å and with a grid spacing of 1 Å, centered around K194-L198.

### MD Simulations

In preparation for MD simulations in explicit solvent, each protein–ligand complex was solvated in a truncated octahedral water box using the TIP3P water model with a minimum distance of 26.0 Å between the solute and the box edge. Na^+^ and Cl^–^ ions were added to balance the charges. All of the preparatory steps were conducted using the program *tLEaP*. Three independent MD runs of 800 ns were performed using AMBER20,^55^ with the ff14SB^56^ force field for the protein, TIP3P^57^ for the solvent, and GAFF^58^ for the small molecules. Ligand mol2 files were processed with antechamber^59^ and parmchk2 to compute AM1-BCC charges.^60^ Amber coordinate and topology files were generated using *tLEaP* from AmberTools, upon combining the processed ligand mol2 file with the apo protein structure. A multistep protocol was adopted for minimizing and equilibrating each complex, consisting of unrestrained minimization of 2000 steps using the steepest descent method during the first 500 steps to handle large forces and the conjugate gradient method during the succeeding 1500 steps. A cutoff distance of 10 Å for nonbonded interactions was used. The system underwent 20 ps of restrained NVT equilibration at 298 K, with a 2 fs time step, using a Langevin thermostat with a damping coefficient of 1 ps^–1^ and harmonic restraints (*k* = 1 kcal/mol/Å^2^) applied to all non-hydrogen atoms except water molecules and ions. This was followed by a 1 ns restrained NPT equilibration at 298 K, using the same Langevin thermostat and the Monte Carlo barostat, with harmonic restraints. A further 1 ns unrestrained *NPT* equilibration was then performed using the same Langevin thermostat and MC barostat, during which harmonic restraints were removed. The production runs consisted of 800 ns of NPT simulations at 298 K with a 2 fs time step, using the Langevin thermostat and MC barostat to maintain pressure at 1 atm. Nonbonded interactions were calculated with a 10 Å cutoff, and long-range electrostatics were treated using the particle-mesh Ewald method, with trajectory data saved every 10 ps for analyses and viewed using PyMol, VMD,^61^ or ChimeraX.^62^ Trajectory analysis was performed using the cpptraj^63^ program.

### Protein–DNA Constructs

PR65 with ybbR tags at the N- and C-termini was conjugated to CoA-modified DNA oligos (biomers) in a 50 μL Sfp-synthase reaction buffer using 10 μM protein, 10 μM Sfp-synthase, and 20 μM CoA oligos. The reaction was kept for overnight at *RT*. Next, the reaction mixture was purified by size exclusion chromatography using a Superdex S200 10–300 equilibrated in 50 mM Tris–HCl, 150 mM NaCl, 1 mM DTT.^27^

DNA handles of size 600 bp were PCR amplified from λ-DNA (Jena Bioscience) using a triple biotinylated primer, a triple digoxigenin modified primer, and a primer with a basic site (biomers). A standard PCR was performed using Q5 master mix DNA polymerase (NEB) at an annealing temperature of 60 °C and an elongation temperature of 68 °C. This PCR produces DNA handles with 5′-overhangs complementary to the CoA-modified oligos. The DNA handles were next purified from agarose gel extraction followed by a QIAquick PCR purification protocol. Different dilutions of oligos-conjugated PR65 was mixed with 200 ng of DNA handles, and the reaction was checked by 1% agarose gel to find the optimum concentration for the DNA–oligos hybridization. Before optical tweezer measurements, the samples were prepared from the fresh hybridization reaction on the same day.

### Optical Tweezers Experiments

Streptavidin and antidigoxigenin coated beads (2 μm) were purchased from Spherotech and stored at 4 °C until use. 10 ng of PR65 constructs was mixed with 1 μL of antidigoxigenin beads in 10 μL of Tris–HCl buffer (50 mM Tris–HCl pH 7.5, 150 mM NaCl, 2 mM DTT). The reaction mixture was then incubated at 4 °C for 30 min. Next, the protein-coated antidigoxigenin beads were dissolved in 450 μL of Tris–HCl buffer for optical tweezers experiments. Optical tweezers measurements for without the SMAP condition (Figure 1) were done in Tris–HCl buffer with 2% DMSO. For measurements done in the presence of SMAP, 10 μM SMAP was dissolved in 2% DMSO and Tris–HCl buffer. Constant velocity experiments at 100 nm/s were performed on a dual trap optical tweezers instrument (C-trap from Lumicks). Tethers were formed by bringing PR65 construct-coated and streptavidin beads in proximity. The protein was stretched and relaxed by moving one of the traps. The data was acquired at 78 kHz and averaged to 100 Hz.

### Data Analysis

To confirm that the data corresponded to a single tether, the following checks were performed: the total measured unfolded length was compared with the expected length and tether breakage in a single rupture event. The quantification of unfolding forces, contour lengths, and refolding forces was done from force extension data, using a custom-built Python code. Unfolding and refolding forces of each transition were determined as the average force before a transition of a minimum 10 nm size. The absolute contour lengths (*L*_c_) were quantified from Worm-like chain (WLC) fitting of the force extension curves. The persistence lengths of the DNA (30 nm) and protein (0.5 nm) and the stretch modulus of DNA (400–600 pN) and the protein (300 pN) were fixed parameters in the WLC model.

### Statistical Analysis

The statistical significance of differences in unfolding and refolding force distributions between experimental conditions was calculated using two samples assuming unequal variance *t*-Test. Test results are mentioned as *p* values in the main text. In box charts, whiskers indicate 90% and 10% extreme values, the inner line represents the median, the length of the box indicates the interquartile range, and the inner small square indicates the mean of the population.

## Supplementary Material



## Data Availability

The Python code used for analysis is available from the corresponding author upon reasonable request. All data supporting the findings of this study are available in the main text and Supporting Information figures. The raw data that support the findings of this study are available from the corresponding authors upon reasonable request.

## Notes

Fluorescence spectra of ATUX-8385 and DBK-766, contour length variations and dependency on unfolding force observed in experiments, distribution of contour length changes associated with each unfolding transition measured from all the stretching curves of PR65 in the presence of ATUX-8385, stability of ATUX-8385–bound PR65 examined by MD simulations, DBK-766 binding to the outward-facing site on the closed conformation of PR65 induces enhanced fluctuations in PR65 conformations, and results from docking simulations of DT-061 onto extended PR65 (PDF)

## ††

Wellcome Sanger Institute, Generative Genomics, Hinxton, Cambridgeshire, CB10 1SA, U.K
