# Supplementary material for: Small Molecule Activators of Protein Phosphatase 2A Exert Global Stabilizing Effects on the Scaffold PR65
Source: JACS Au. 2026 Apr 20;6(5):2753–65. doi: 10.1021/jacsau.6c00003 (PMC13213488; doi:10.1021/jacsau.6c00003)
Supplement: Supplementary file 1 [file au6c00003_si_001.pdf]

Supporting Information

Small Molecule Activators of Protein Phosphatase 2A Exert Global Stabilising Effects on the Scaffold PR65

Mohsin M. Naqvi[1,11+], Maria Zacharopoulou[1+], Satyaki Saha[2,3+], Anupam Banerjee[2,3], Sema Z. Yilmaz[4], Vanda Sunderlikova[5,6], Chris M. Johnson[7], Janet R. Kumita[1], Shang-Hua Yang[8], Reuven Gordon[9], Michael Ohlmeyer, Sander Tans[5,6], Mert Gur[4], Ivet Bahar[2,3*], Laura S. Itzhaki[1*]

Department of Pharmacology, University of Cambridge, Tennis Court Road, Cambridge CB2 1PD, UK

[2] Laufer Center for Physical and Quantitative Biology, Stony Brook University, NY 11794, USA

[3] Department of Biochemistry and Cell Biology, Renaissance School of Medicine, Stony Brook University, NY 11794, USA

[4] Department of Computational and Systems Biology, School of Medicine, University of Pittsburgh, Pittsburgh, PA 15260, USA

[5] AMOLF, Science Park 104, 1098 XG Amsterdam, the Netherlands

[6] Department of Bionanoscience, Kavli Institute of Nanoscience Delft, Delft University of Technology, 2629 HZ Delft, The Netherlands

[7] MRC Laboratory of Molecular Biology, Francis Crick Avenue, Cambridge, CB2 0QH, UK

[8] Department of Electrical Engineering, National Tsing Hua University, Hsinchu, 30013, Taiwan

[9] Department of Electrical and Computer Engineering, University of Victoria, Victoria, BC V8P 5C2, Canada

[10] Atux Iskay LLC, Plainsboro, New Jersey, NJ, 08536, USA

[11] Current address: Wellcome Sanger Institute, Generative Genomics, Hinxton, Cambridgeshire, CB10 1SA, UK

+These authors contributed equally

*Corresponding authors: lsi10@cam.ac.uk (LS Itzhaki), bahar@laufercenter.org (I Bahar)

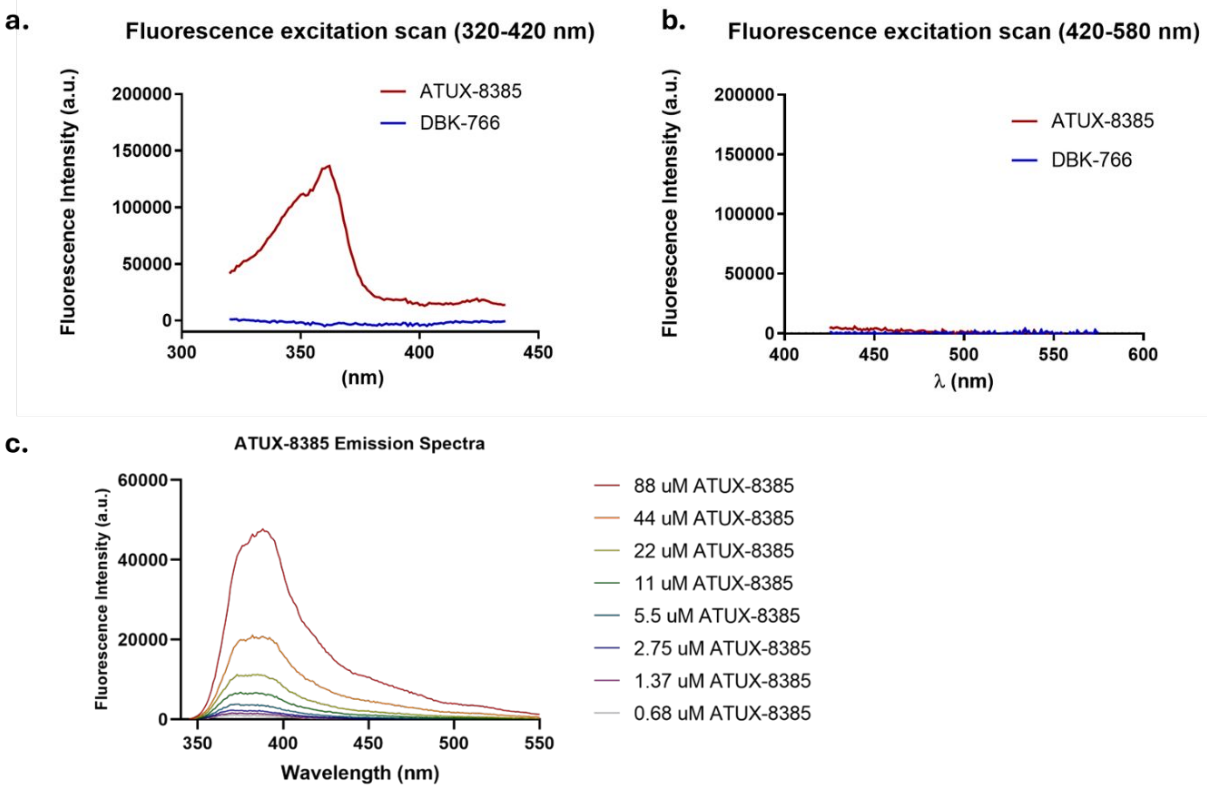

Fig. S1: Fluorescence spectra of ATUX-8385 and DBK-766. Fluorescence excitation scans from 320-420 nm **(a)** and 420-580 nm **(b)** of ATUX-8385 and DBK-766 show that ATUX-8385 has an excitation maximum at ~350 nm, whereas DBK-766 is not fluorescent. **c)** Fluorescence emission spectrum of ATUX-8385 upon excitation at 290 nm.

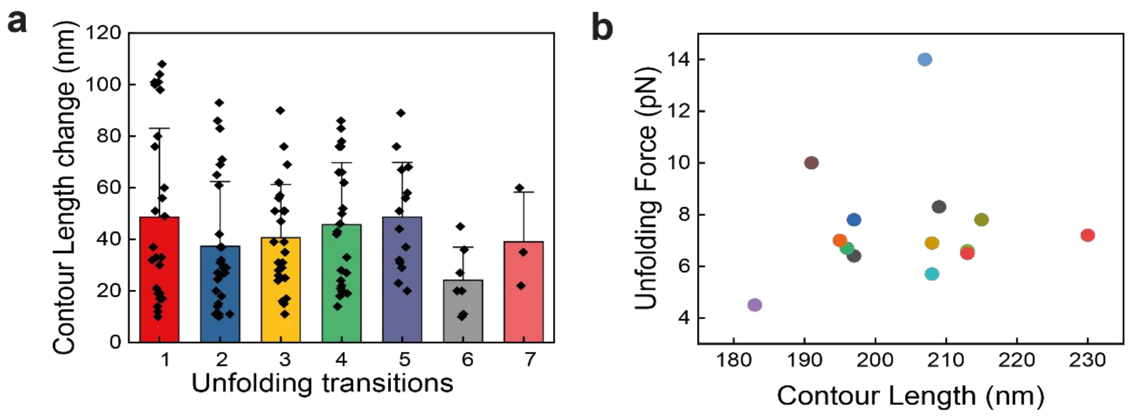

Fig. S2. Contour length variations and dependency on unfolding force observed in experiments. a) Distribution of contour length changes associated with individual unfolding transitions measured from all the stretching curves of PR65 in the absence of SMAP. **b)** Maximum unfolding force vs absolute contour length of the fully unfolded molecule measured from the first pulls of all the PR65 molecules in the absence of SMAP.

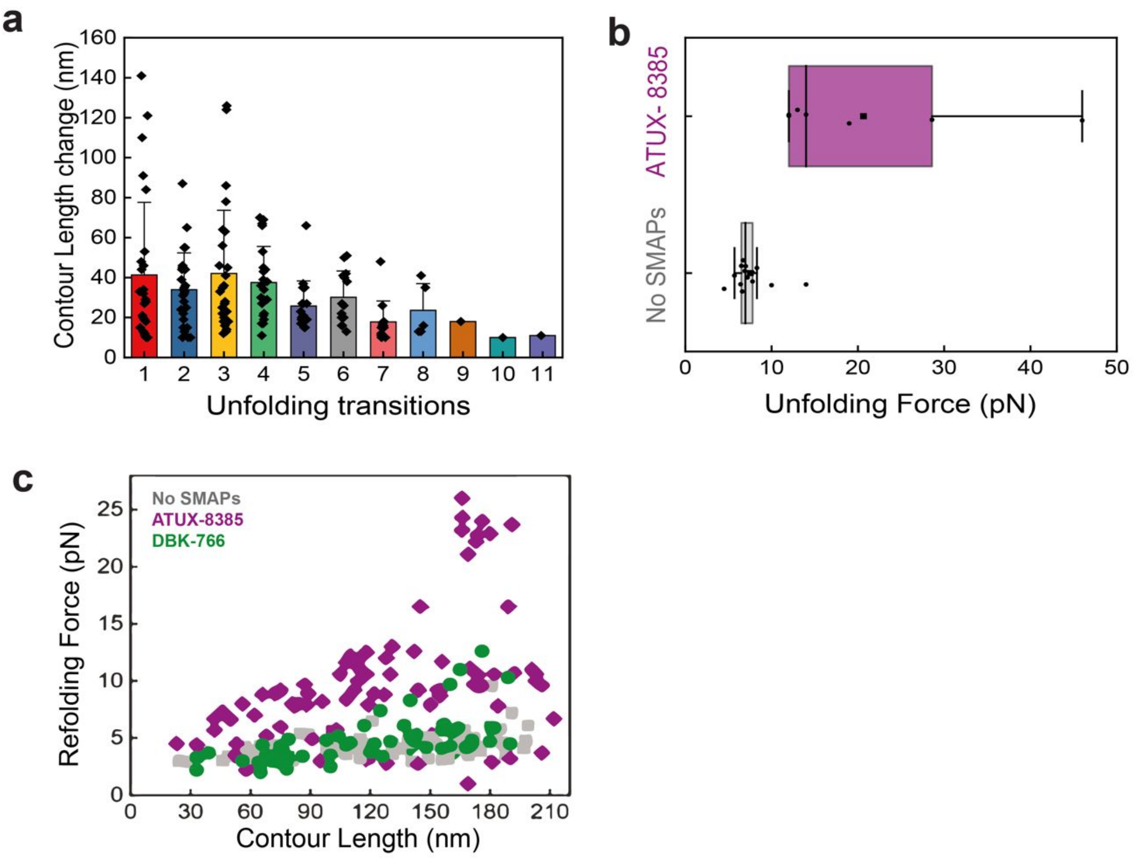

Fig. S3. a) Distribution of contour length changes associated with each unfolding transition measured from all the stretching curves of PR65 in the presence of ATUX-8385. **b)** Box plots showing distribution of maximum unfolding force of the first pulls for PR65 with (*purple*) and without (*gray*) SMAP. **(c)** Refolding force *vs* absolute contour length (L_c) of each intermediate state observed during relaxation of apo PR65 (*gray squares*), and PR65 bound to ATUX-8385 (*purple diamonds*) and DBK-766 (*green circles*).

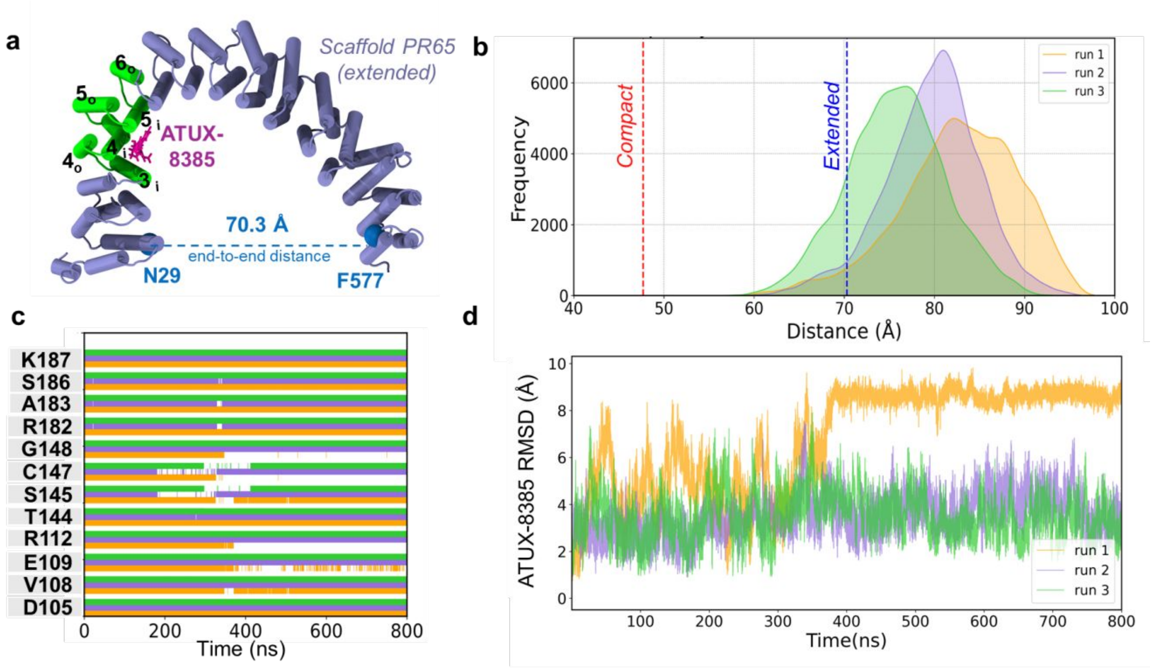

Fig. S4. Stability of ATUX-8385–bound PR65 examined by MD simulations. a) Binding pose of ATUX-8385 to PR65. The diagram shows the initial conformation used in MD simulations. **b)** Histogram of end-to-end distances sampled during MD simulations. The histograms for the three runs are shown in different colors. **c)** Time evolution of ATUX-8385 binding with coordinating PR65 residues. Coordinating residues are those making atom-atom contacts within a cutoff distance of 5 Å across three independent runs, represented by *orange*, *purple*, and *green*, respectively. Blank regions refer to cases where those particular residues were more than 5 Å away from ATUX-8385. **d)** ATUX-8385 RMSD from MD snapshots. In run1, ATUX-8385 undergoes a rotational motion at about 400 ns and remains bound at the same site, but with a different orientation.

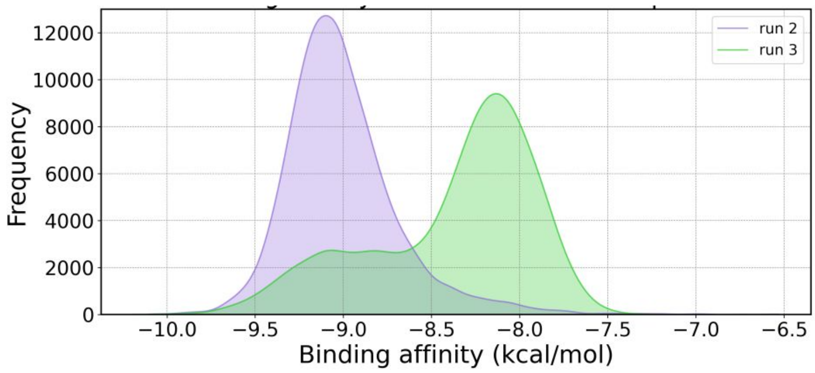

Fig. S5. Distribution of DBK-766 binding energies observed for DBK-766 bound to the outer site in PR65. Results from two runs (*runs 2 and 3*) are shown, in which DBK-766 remained bound to PR65. The binding affinity is calculated using DBK-766 dissociated from PR65 in *run 1.*

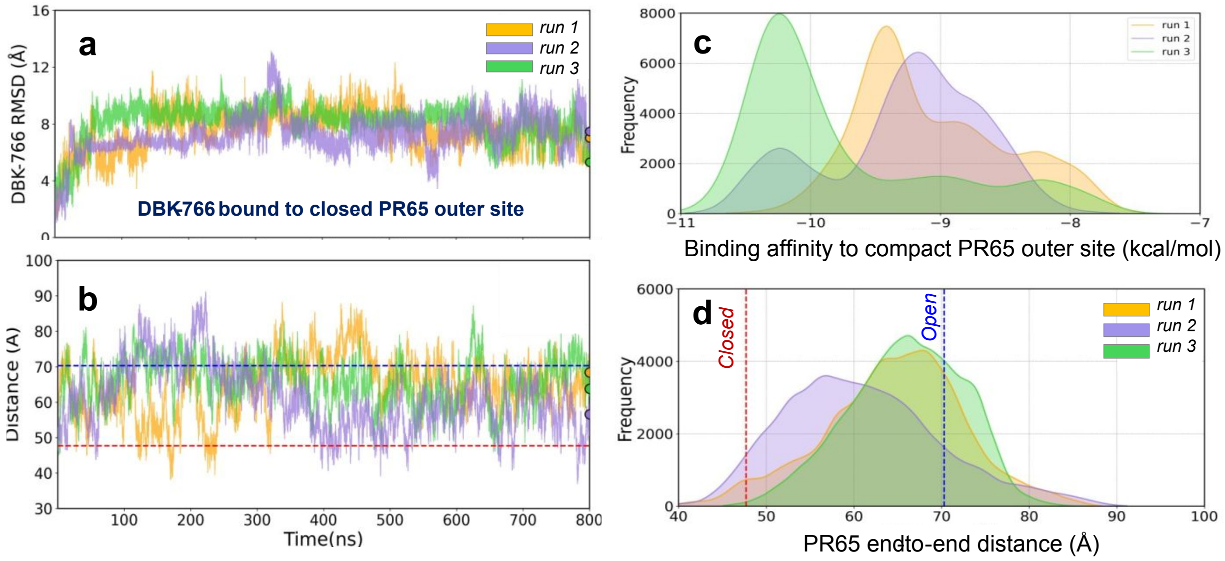

Fig S6. DBK-766 binding to the outward-facing site on the closed conformation of PR65 induces enhanced fluctuations in PR65 conformations. (a) Fluctuations in the position of DBK-766 bound to the outward-facing site of PR65 shown in **Fig 7a** when PR65 is in the closed state**.** The RMSD profile indicates rearrangements within that site but not any significant dislocation. **(b)** Large fluctuations in PR65 end-to-end distance as a function of time; note that the structure tends to sample conformations close to the open state, although the simulations were initiated from the closed state. **(c)** Histogram of DBK-766 binding affinity observed during the three MD runs. Strong binding is observed in general. **(d)** Histogram of the end-to-end distance shows variations from 40 to 90 Å, suggesting a destabilizing effect exerted by DBK-766. The *dashed vertical lines* indicate the end-to-end distances corresponding to the experimentally resolved open and closed states.

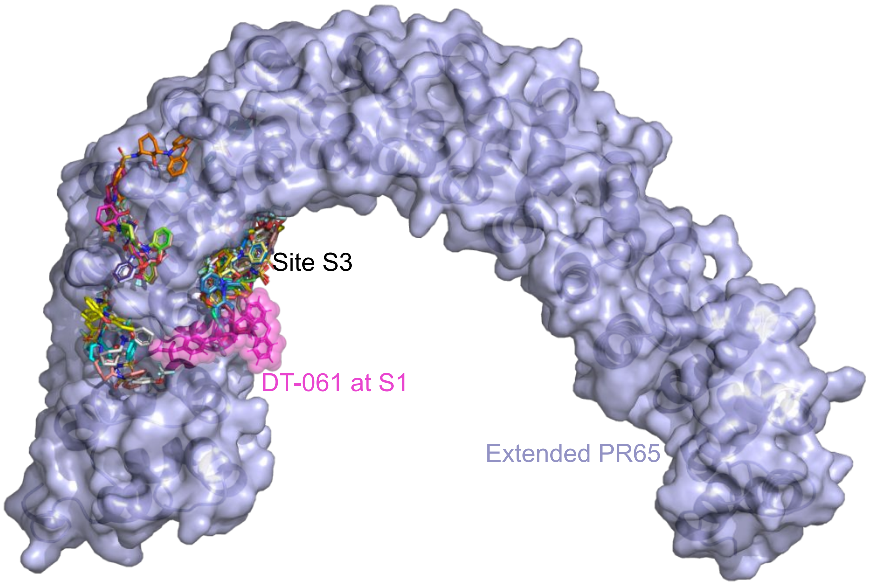

Fig. S7. Results from docking simulations of DT-061 onto extended PR65. Multiple binding poses are shown (multicolor sticks). Site S3 is distinguished by its high affinity to bind DT-061 in multiple runs. For comparison DT-061 *(pink sticks and shade*) is also shown at the site (S1) resolved by cryo-EM for the trimeric PP2A. The Vina binding affinity of the best docked pose at S3 is -7.88 kcal/mol, and that of PRODIGY-LIG is -9.48 kcal/mol.
